# Supplementary material for: Influenza A Hemagglutinin Passage Bias Sites and Host Specificity Mutations
Source: Cells. 2019 Aug 22;8(9):958. doi: 10.3390/cells8090958 (PMC6770435; doi:10.3390/cells8090958)
Supplement: Supplementary file 1 [file cells-08-00958-s001.pdf]

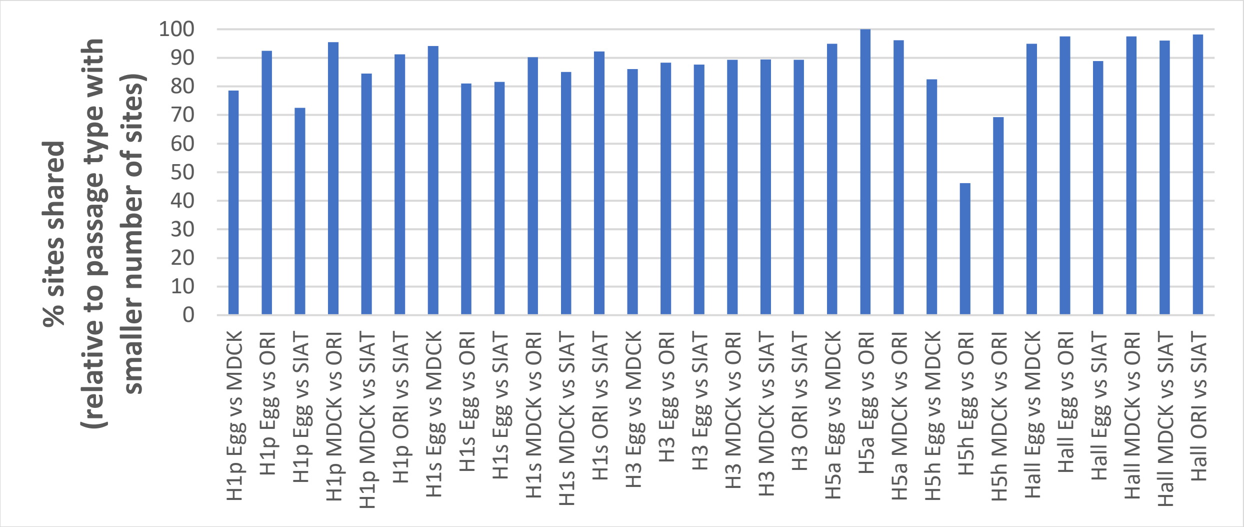

Suppl. Figure 1. Overlap of passage bias sites between different passage types

		**MDCK**	**ORI**	**SIAT**	**EGG**
H1N1 HA	Human	9845	3174	427	755
H3N2 HA	Human	9440	5008	4291	609

Suppl. Table 1. Updated passage data set for seasonal influenza A site pair analysis

H1p absolute numbering	H1 absolute numbering	H1 numbering	H3 absolute numbering	H3 numbering	H5 absolute numbering	H5 numbering	Geometric Mean of Odds Ratios from subtype passage combinations	#occurrences (in 18 possible subtype passage combinations)	#ssdp H1p	#ssdp H1s	#ssdp H3
1	1	-17	1	-16	1	-16	1	0	0	0	0
2	2	-16	2	-15	2	-15	3.01	9	1	0	1
3	3	-15	gap	gap	3	-14	4.86	14	8	2	0
gap	gap	gap	gap	gap	4	-13	NA	NA	NA	NA	NA
4	4	-14	3	-14	5	-12	5.4	13	0	2	0
5	5	-13	4	-13	6	-11	6.18	13	1	0	0
6	6	-12	5	-12	7	-10	28.28	16	2	0	0
7	7	-11	6	-11	8	-9	9.19	13	0	0	0
8	8	-10	7	-10	9	-8	5.06	10	2	0	0
9	9	-9	8	-9	10	-7	12.59	15	1	0	1
10	10	-8	9	-8	11	-6	4.92	10	0	0	0
11	11	-7	10	-7	12	-5	12.53	16	0	0	2
12	12	-6	11	-6	13	-4	10.95	16	0	0	0

13	13	-5	12	-5	14	-3	10.44	13	2	0	2
14	14	-4	13	-4	15	-2	12.89	15	1	0	0
15	15	-3	14	-3	16	-1	12.33	13	2	1	1
16	16	-2	15	-2	gap	gap	13.36	15	0	0	0
17	17	-1	16	-1	gap	gap	4.47	10	0	0	0
gap	gap	gap	17	1	gap	gap	NA	NA	NA	NA	NA
gap	gap	gap	18	2	gap	gap	NA	NA	NA	NA	NA
gap	gap	gap	19	3	gap	gap	NA	NA	NA	NA	NA
gap	gap	gap	20	4	gap	gap	NA	NA	NA	NA	NA
gap	gap	gap	21	5	gap	gap	NA	NA	NA	NA	NA
gap	gap	gap	22	6	gap	gap	NA	NA	NA	NA	NA
gap	gap	gap	23	7	gap	gap	NA	NA	NA	NA	NA
gap	gap	gap	24	8	gap	gap	NA	NA	NA	NA	NA
gap	gap	gap	25	9	gap	gap	NA	NA	NA	NA	NA
gap	gap	gap	26	10	gap	gap	NA	NA	NA	NA	NA
18	18	1	27	11	17	1	1.36	3	0	0	0
19	19	2	28	12	18	2	3.15	12	0	0	0
20	20	3	29	13	19	3	2.2	6	0	0	0
21	21	4	30	14	20	4	1	0	0	0	0
22	22	5	31	15	21	5	2.79	7	1	0	0
23	23	6	32	16	22	6	1	0	0	0	0
24	24	7	33	17	23	7	1.34	4	1	0	0
25	25	8	34	18	24	8	1.12	2	0	0	0
26	26	9	35	19	25	9	2.3	6	2	0	0
27	27	10	36	20	26	10	1.08	1	0	0	0
28	28	11	37	21	27	11	2.38	4	0	0	0
29	29	12	38	22	28	12	1.64	4	0	0	0
30	30	13	39	23	29	13	1	0	0	0	0
31	31	14	40	24	30	14	2.39	9	0	0	0
32	32	15	41	25	31	15	3.72	9	0	0	0
33	33	16	42	26	32	16	1.36	5	0	0	0
34	34	17	43	27	33	17	1.06	1	0	0	0
35	35	18	44	28	34	18	1	0	0	0	0
36	36	19	45	29	35	19	1.68	7	1	1	0
37	37	20	46	30	36	20	1.89	4	0	0	0
38	38	21	47	31	37	21	2.93	6	2	0	0
39	39	22	48	32	38	22	2.16	8	0	0	0
40	40	23	49	33	39	23	2.47	6	0	0	0
41	41	24	50	34	40	24	1.69	6	0	0	1
42	42	25	51	35	41	25	1.16	2	0	0	0
43	43	26	52	36	42	26	1.11	2	0	0	0
44	44	27	53	37	43	27	1.2	2	0	0	0
45	45	28	54	38	44	28	1.04	1	0	0	0
46	46	29	55	39	45	29	1	0	0	0	0
47	47	30	56	40	46	30	1.92	7	0	0	0
48	48	31	57	41	47	31	1.38	4	1	0	0

49	49	32	58	42	48	32	1.25	3	0	0	0
50	50	33	59	43	49	33	1.05	1	0	0	0
51	51	34	60	44	50	34	1	0	0	0	0
52	52	35	61	45	51	35	7.08	13	0	1	0
53	53	36	62	46	52	36	9.02	14	0	0	0
54	54	37	63	47	53	37	2.32	6	0	0	0
55	55	38	64	48	54	38	3.3	9	2	0	0
56	56	39	65	49	55	39	2.22	9	0	0	0
57	57	40	66	50	56	40	4.04	8	0	0	0
58	58	41	67	51	57	41	1.07	1	1	0	0
59	59	42	68	52	58	42	1	0	0	0	0
60	60	43	69	53	59	43	15.26	16	1	0	1
61	61	44	70	54	60	44	1.07	1	1	0	0
62	62	45	gap	gap	61	45	7.99	11	1	0	0
63	63	46	71	55	62	46	1.17	3	0	0	0
64	64	47	72	56	63	47	2.89	10	0	0	0
65	65	48	73	57	64	48	6.46	12	0	0	0
66	66	49	74	58	65	49	1.75	4	0	0	0
67	67	50	75	59	66	50	1	0	0	0	1
68	68	51	76	60	67	51	2.2	7	1	0	0
69	69	52	77	61	68	52	1	0	0	0	0
70	70	53	78	62	69	53	7.26	9	0	0	0
71	71	54	79	63	70	54	4.75	11	0	0	0
72	72	55	80	64	71	55	1	0	0	0	0
73	73	56	81	65	72	56	2.83	11	0	0	0
74	74	57	82	66	73	57	2.27	9	0	0	0
75	75	58	83	67	74	58	1.98	6	0	0	0
76	76	59	84	68	75	59	1	0	0	0	0
77	77	60	85	69	76	60	1.47	3	0	0	0
78	78	61	86	70	77	61	4.69	9	2	0	0
79	79	62	87	71	78	62	1	0	0	0	0
80	80	63	88	72	79	63	1	0	0	0	0
81	81	64	89	73	80	64	1	0	0	0	0
82	82	65	90	74	81	65	1	0	0	0	0
83	83	66	91	75	82	66	4.88	12	0	0	3
84	84	67	92	76	83	67	1	0	0	0	0
85	85	68	93	77	84	68	5.9	12	1	0	0
86	86	69	94	78	85	69	21.67	14	0	0	1
87	87	70	95	79	86	70	1.4	5	0	0	0
88	88	71	96	80	87	71	35.95	17	0	0	0
89	89	72	97	81	88	72	6.27	14	0	0	0
90	90	73	gap	gap	89	73	4.93	10	0	0	0
91	91	74	98	82	90	74	18.62	15	0	0	0
92	92	75	99	83	91	75	3.8	7	0	0	1
93	93	76	100	84	92	76	1	0	0	0	0
94	94	77	101	85	93	77	1.45	5	0	0	0

95	95	78	102	86	94	78	1.28	3	0	0	0
96	96	79	103	87	95	79	4.16	7	1	0	0
97	97	80	104	88	96	80	4.21	8	1	0	0
98	98	81	105	89	97	81	1	0	0	0	0
99	99	82	106	90	98	82	2.94	9	0	1	0
100	100	83	107	91	99	83	6.08	15	0	0	0
101	101	84	108	92	100	84	9.49	13	2	0	0
102	102	85	109	93	101	85	2.45	9	0	0	0
103	103	86	110	94	102	86	5.6	14	0	0	0
104	104	87	111	95	103	87	6.41	9	0	0	5
105	105	88	112	96	104	88	1.45	7	0	0	0
106	106	89	gap	gap	105	89	2.83	12	0	0	6
107	107	90	113	97	106	90	1	0	0	0	0
108	108	91	114	98	107	91	1	0	0	0	0
109	109	92	115	99	108	92	1	0	0	0	0
110	110	93	116	100	109	93	1.62	3	0	0	0
111	111	94	117	101	110	94	18.65	18	0	1	0
112	112	95	118	102	111	95	2.51	7	0	0	0
113	113	96	119	103	112	96	4.41	14	0	1	0
114	114	97	120	104	113	97	2.73	9	0	0	0
115	115	98	121	105	114	98	1.71	4	0	0	0
116	116	99	122	106	115	99	2.04	7	0	1	0
117	117	100	123	107	116	100	1.47	4	0	0	0
118	118	101	124	108	117	101	1	0	0	0	0
119	119	102	125	109	118	102	1.36	3	0	0	0
120	120	103	gap	gap	119	103	1	0	1	0	0
121	121	104	gap	gap	120	104	2.18	8	0	1	0
122	122	105	gap	gap	121	105	1.75	7	0	0	0
123	123	106	126	110	122	106	2.45	6	1	0	0
124	124	107	127	111	123	107	3.45	12	0	1	0
125	125	108	128	112	124	108	1.44	4	0	0	0
126	126	109	129	113	125	109	1.42	4	0	0	0
127	127	110	130	114	126	110	2.43	9	0	0	0
128	128	111	131	115	127	111	1.81	8	1	0	0
129	129	112	132	116	128	112	1.4	5	0	0	1
130	130	113	133	117	129	113	2.16	6	0	0	0
131	131	114	134	118	130	114	1.27	4	0	0	0
132	132	115	135	119	131	115	2.78	8	0	0	0
133	133	116	136	120	132	116	1.55	5	0	0	1
134	134	117	137	121	133	117	1.11	2	0	0	0
135	135	118	138	122	134	118	4.16	5	0	0	1
136	136	119	139	123	135	119	7.35	12	1	0	0
137	137	120	140	124	gap	gap	3.39	7	1	0	0
138	138	121	141	125	136	120	24.19	18	0	0	0
139	139	122	142	126	137	121	5.92	9	0	0	1
140	140	123	143	127	138	122	1	0	0	0	0

141	141	124	144	128	139	123	4.71	11	1	0	1
142	142	125	145	129	140	124	7.44	14	2	3	1
143	143	126	146	130	141	125	1.43	4	0	0	1
144	144	127	147	131	142	126	11.42	16	71	1	1
145	145	128	148	132	143	127	9.25	17	1	0	0
146	146	129	149	133	144	128	13.47	15	8	0	0
147	gap	gap	gap	gap	145	129	4.82	11	0	0	0
148	147	130	150	134	146	130	1	0	0	0	0
149	148	131	151	135	147	131	7.52	12	0	0	1
150	149	132	152	136	148	132	1.76	5	0	0	0
151	150	133	153	137	149	133	10.68	13	2	0	1
152	151	134	154	138	150	134	3.22	9	0	0	28
153	152	135	155	139	151	135	1	0	0	0	0
154	153	136	156	140	152	136	11.92	16	1	0	11
155	154	137	157	141	153	137	3.91	11	1	0	0
156	155	138	158	142	154	138	12.79	16	0	0	7
157	156	139	159	143	155	139	4.28	7	0	3	0
158	157	140	160	144	156	140	81.24	18	0	2	2
159	158	141	161	145	157	141	19.42	17	0	0	5
160	159	142	162	146	158	142	3.71	8	0	0	0
161	160	143	163	147	159	143	1	0	0	0	0
162	161	144	164	148	160	144	1.22	3	0	1	0
163	162	145	165	149	161	145	7.97	14	1	0	0
164	163	146	166	150	162	146	1.18	2	0	0	0
165	164	147	167	151	163	147	1	0	0	1	0
166	165	148	168	152	164	148	2.72	9	1	0	0
167	166	149	169	153	165	149	1	0	0	0	0
168	167	150	170	154	166	150	3.88	7	0	0	0
169	168	151	171	155	167	151	8.31	15	3	4	0
170	169	152	172	156	168	152	11.11	12	2	0	37
171	170	153	173	157	169	153	3.36	7	1	1	0
172	171	154	174	158	170	154	12.97	15	3	0	2
173	172	155	175	159	171	155	31.17	17	2	0	1
174	173	156	176	160	172	156	10.03	17	0	0	118
175	174	157	177	161	173	157	1.13	2	0	0	1
176	175	158	178	162	174	158	1.95	6	0	1	0
177	176	159	179	163	175	159	12.41	14	0	0	0
178	177	160	180	164	176	160	2.83	10	2	2	1
179	178	161	181	165	177	161	3.56	12	19	0	0
180	179	162	182	166	178	162	8.68	16	4	0	0
181	180	163	183	167	179	163	3.97	10	20	0	1
182	181	164	184	168	180	164	2.61	6	0	0	0
183	182	165	185	169	181	165	11.88	15	0	0	0
184	183	166	186	170	182	166	1	0	0	0	0
185	184	167	187	171	183	167	4.21	13	0	0	0
186	185	168	188	172	184	168	5.67	11	1	0	0

187	186	169	189	173	185	169	16.16	15	0	0	0
188	187	170	190	174	186	170	2.18	9	1	0	0
189	188	171	191	175	187	171	2.34	11	1	0	0
190	189	172	192	176	188	172	1.04	1	2	0	0
191	190	173	193	177	189	173	1.29	3	0	0	0
192	191	174	194	178	190	174	2.5	7	0	0	0
193	192	175	195	179	191	175	9.09	14	0	0	0
194	193	176	196	180	192	176	1	0	0	0	0
195	194	177	197	181	193	177	2.04	6	0	0	0
196	195	178	198	182	194	178	2.9	10	0	0	0
197	196	179	199	183	195	179	1.65	3	0	0	19
198	197	180	200	184	196	180	1.05	1	0	0	0
199	198	181	201	185	197	181	1.28	2	0	1	0
200	199	182	202	186	198	182	6.03	10	24	3	114
201	200	183	203	187	199	183	4.11	14	5	0	0
202	201	184	204	188	200	184	20.84	17	1	2	5
203	202	185	205	189	201	185	23.25	16	0	45	0
204	203	186	206	190	202	186	12.73	11	30	0	26
205	204	187	207	191	203	187	1.28	2	0	2	0
206	205	188	208	192	204	188	9.09	15	0	0	0
207	206	189	209	193	205	189	11.92	17	13	0	5
208	207	190	210	194	206	190	7.59	13	71	0	145
209	208	191	211	195	207	191	1.45	4	0	0	1
210	209	192	212	196	208	192	8.4	14	0	1	2
211	210	193	213	197	209	193	5.69	12	0	0	1
212	211	194	214	198	210	194	19.9	14	1	0	1
213	212	195	215	199	211	195	4.35	13	0	0	0
214	213	196	216	200	212	196	1.89	6	2	0	0
215	214	197	217	201	213	197	1.63	5	0	0	3
216	215	198	218	202	214	198	3.36	10	0	0	0
217	216	199	219	203	215	199	3.76	11	0	0	36
218	217	200	220	204	216	200	1.9	8	0	0	0
219	218	201	221	205	217	201	2.8	8	5	0	0
220	219	202	222	206	218	202	1.84	6	0	0	0
221	220	203	223	207	219	203	2.18	6	0	0	1
222	221	204	224	208	220	204	7.33	12	1	0	1
223	222	205	225	209	221	205	1.34	3	1	0	0
224	223	206	226	210	222	206	3.54	7	0	0	0
225	224	207	227	211	223	207	2.6	8	0	1	1
226	225	208	228	212	224	208	4.21	11	5	0	0
227	226	209	229	213	225	209	4.68	11	0	0	0
228	227	210	230	214	226	210	6.14	14	1	0	0
229	228	211	231	215	227	211	1	0	0	0	0
230	229	212	232	216	228	212	3.11	9	0	0	0
231	230	213	233	217	229	213	2.08	11	1	0	0
232	231	214	234	218	230	214	3.62	13	15	0	0

233	232	215	235	219	231	215	11.51	12	6	0	64
234	233	216	236	220	232	216	1.92	4	0	0	0
235	234	217	237	221	233	217	3.19	11	10	0	6
236	235	218	238	222	234	218	9.97	11	1	0	1
237	236	219	239	223	235	219	4.53	14	2	0	2
238	237	220	240	224	236	220	1.06	1	5	6	0
239	238	221	241	225	237	221	6.65	12	89	6	64
240	239	222	242	226	238	222	16.05	10	163	3	10
241	240	223	243	227	239	223	10.73	13	16	0	6
242	241	224	244	228	240	224	4.4	7	6	0	0
243	242	225	245	229	241	225	1.75	4	0	0	0
244	243	226	246	230	242	226	3.7	12	1	0	3
245	244	227	247	231	243	227	2.94	9	0	0	0
246	245	228	248	232	244	228	1.97	4	0	0	0
247	246	229	249	233	245	229	1.2	3	0	0	0
248	247	230	250	234	246	230	1	0	0	0	0
249	248	231	251	235	247	231	1.75	8	0	0	0
250	249	232	252	236	248	232	1.75	7	1	0	0
251	250	233	253	237	249	233	2.05	6	1	0	0
252	251	234	254	238	250	234	5.56	14	0	0	0
253	252	235	255	239	251	235	20.71	15	0	0	1
254	253	236	256	240	252	236	1.81	10	0	0	0
255	254	237	257	241	253	237	2.48	6	0	0	1
256	255	238	258	242	254	238	5.02	13	0	0	0
257	256	239	259	243	255	239	1.76	4	0	0	0
258	257	240	260	244	256	240	5.96	14	0	0	0
259	258	241	261	245	257	241	1.22	2	0	0	0
260	259	242	262	246	258	242	2.41	6	0	0	31
261	260	243	263	247	259	243	1.89	4	0	0	0
262	261	244	264	248	260	244	5.6	14	0	0	4
263	262	245	265	249	261	245	1	0	0	0	0
264	263	246	266	250	262	246	1	0	0	0	0
265	264	247	267	251	263	247	1.5	5	0	0	0
266	265	248	268	252	264	248	2.64	9	0	0	0
267	266	249	269	253	265	249	1.84	7	0	0	0
268	267	250	270	254	266	250	1	0	0	0	0
269	268	251	271	255	267	251	4.18	10	1	1	0
270	269	252	272	256	268	252	3.53	10	0	0	0
271	270	253	273	257	269	253	1	0	0	0	0
272	271	254	274	258	270	254	1	0	0	0	0
273	272	255	275	259	271	255	2.28	8	1	0	0
274	273	256	276	260	272	256	5.02	13	1	0	1
275	274	257	gap	gap	273	257	8.3	10	0	1	0
276	275	258	277	261	274	258	5.07	11	0	0	0
277	276	259	278	262	275	259	9.43	16	2	0	1
278	277	260	279	263	276	260	4.45	10	2	0	0

279	278	261	280	264	277	261	7.82	12	0	0	1
280	279	262	281	265	278	262	3.01	10	0	0	0
281	280	263	282	266	279	263	3.18	11	1	1	0
282	281	264	283	267	280	264	1.72	5	0	0	0
283	282	265	284	268	281	265	2.24	8	1	2	0
284	283	266	285	269	282	266	10.1	17	0	0	2
285	284	267	286	270	283	267	1.5	4	0	0	0
286	285	268	287	271	284	268	3.13	13	1	0	0
287	286	269	288	272	285	269	4.5	15	0	0	0
288	287	270	289	273	286	270	12.36	14	2	0	0
289	288	271	290	274	287	271	3.56	10	0	0	0
290	289	272	291	275	288	272	14.31	17	0	1	0
291	290	273	292	276	289	273	18.02	16	1	0	0
292	291	274	293	277	290	274	1	0	0	0	0
293	292	275	294	278	291	275	9.91	15	0	0	0
294	293	276	295	279	292	276	2.87	12	0	0	0
295	294	277	296	280	293	277	16.38	14	0	0	0
296	295	278	297	281	294	278	1	0	0	0	0
297	296	279	298	282	295	279	1.43	5	0	0	0
298	297	280	299	283	296	280	1.73	6	0	0	0
299	298	281	300	284	297	281	2.75	10	0	0	0
300	299	282	301	285	298	282	11.54	14	0	0	0
301	300	283	302	286	299	283	1.3	3	0	0	0
302	301	284	303	287	300	284	1.12	2	0	0	0
303	302	285	304	288	301	285	4.01	11	0	0	0
304	303	286	305	289	302	286	2.08	11	1	0	0
305	304	287	306	290	303	287	3.23	9	0	0	1
306	305	288	307	291	304	288	2.07	9	0	0	1
307	306	289	308	292	305	289	2.84	10	0	0	0
308	307	290	309	293	306	290	1	0	0	0	0
309	308	291	310	294	307	291	1.13	2	0	0	0
310	309	292	311	295	308	292	1.16	2	0	0	0
311	310	293	312	296	309	293	1.73	5	0	0	0
312	311	294	313	297	310	294	2.48	10	0	0	0
313	312	295	314	298	311	295	2.04	6	0	0	0
314	313	296	315	299	312	296	3.38	9	0	0	0
315	314	297	316	300	313	297	2.3	9	0	0	0
316	315	298	317	301	314	298	1.12	2	0	0	0
317	316	299	318	302	315	299	1.29	4	0	0	0
318	317	300	319	303	316	300	1	0	0	0	0
319	318	301	320	304	317	301	6.17	14	0	0	0
320	319	302	321	305	318	302	1	0	0	0	0
321	320	303	322	306	319	303	2.27	9	0	0	0
322	321	304	323	307	320	304	4.77	11	0	0	0
323	322	305	324	308	321	305	1	0	0	0	0
324	323	306	325	309	322	306	1.68	6	0	0	1

325	324	307	326	310	323	307	1.71	8	0	0	0
326	325	308	327	311	324	308	1.45	6	0	1	0
327	326	309	328	312	325	309	9.63	17	0	0	1
328	327	310	329	313	326	310	5.95	15	0	0	0
329	328	311	330	314	327	311	1	0	0	0	0
330	329	312	331	315	328	312	1.23	4	2	0	0
331	330	313	332	316	329	313	1.81	6	0	0	0
332	331	314	333	317	330	314	1.68	5	0	0	0
333	332	315	334	318	331	315	1.45	3	0	0	0
334	333	316	335	319	332	316	1	0	0	0	0
335	334	317	336	320	333	317	1.22	4	0	1	0
336	335	318	337	321	334	318	1	0	2	0	0
337	336	319	338	322	335	319	1	0	1	0	0
338	337	320	339	323	336	320	2.59	11	2	0	1
339	338	321	340	324	337	321	1.19	2	0	0	0
340	339	322	341	325	338	322	1.66	8	0	1	0
341	340	323	342	326	339	323	4.17	13	2	0	0
342	341	324	343	327	340	324	2.05	5	0	0	0
gap	gap	gap	gap	gap	341	325	NA	NA	NA	NA	NA
gap	gap	gap	gap	gap	342	326	NA	NA	NA	NA	NA
gap	gap	gap	gap	gap	343	327	NA	NA	NA	NA	NA
gap	gap	gap	gap	gap	344	328	NA	NA	NA	NA	NA
343	342	325	344	328	345	329	2.83	8	1	1	1
344	343	326	345	329	346	330	1.7	6	0	0	0
345	344	327	346	330	347	331	1	0	0	0	0
346	345	328	347	331	348	332	3.03	7	0	2	0
347	346	329	348	332	349	333	1.48	5	5	0	0
348	347	330	349	333	350	334	1.35	4	0	0	0
349	348	331	350	334	351	335	1	0	0	0	0
350	349	332	351	335	352	336	1.66	4	0	0	1
351	350	333	352	336	353	337	1	0	0	0	0
352	351	334	353	337	354	338	1	0	0	0	0
353	352	335	354	338	355	339	1	0	0	0	1
354	353	336	355	339	356	340	1	0	1	0	0
355	354	337	356	340	357	341	1	0	0	0	0
356	355	338	357	341	358	342	1.1	2	0	0	0
357	356	339	358	342	359	343	1	0	0	0	0
358	357	340	359	343	360	344	1	0	0	0	0
359	358	341	360	344	361	345	1.1	2	0	0	0
360	359	342	361	345	362	346	1	0	0	0	0
361	360	343	362	346	363	347	1.8	4	0	0	0
362	361	344	363	347	364	348	6.27	10	1	1	0
363	362	345	364	348	365	349	1.44	4	1	0	0
364	363	346	365	349	366	350	1	0	0	0	0
365	364	347	366	350	367	351	1	0	0	0	0
366	365	348	367	351	368	352	1	0	0	0	0

367	366	349	368	352	369	353	1	0	0	0	0
368	367	350	369	353	370	354	2.25	10	0	0	1
369	368	351	370	354	371	355	1.84	6	0	0	0
370	369	352	371	355	372	356	2.84	7	2	0	2
371	370	353	372	356	373	357	1.52	6	0	0	0
372	371	354	373	357	374	358	1	0	0	0	0
373	372	355	374	358	375	359	1.8	9	0	0	0
374	373	356	375	359	376	360	1.47	5	0	0	0
375	374	357	376	360	377	361	1	0	1	0	0
376	375	358	377	361	378	362	2.84	7	0	0	0
377	376	359	378	362	379	363	1	0	0	0	0
378	377	360	379	363	380	364	1.48	4	4	0	0
379	378	361	380	364	381	365	1.12	2	0	0	0
380	379	362	381	365	382	366	1	0	0	0	0
381	380	363	382	366	383	367	1	0	0	0	0
382	381	364	383	367	384	368	3.34	13	0	0	0
383	382	365	384	368	385	369	3.4	13	0	0	0
384	383	366	385	369	386	370	1	0	0	0	0
385	384	367	386	370	387	371	1	0	1	0	0
386	385	368	387	371	388	372	1	0	0	0	0
387	386	369	388	372	389	373	4.65	12	0	0	1
388	387	370	389	373	390	374	1.04	1	3	0	0
389	388	371	390	374	391	375	3.49	7	0	0	0
390	389	372	391	375	392	376	3.42	12	0	0	0
391	390	373	392	376	393	377	3.15	8	1	0	0
392	391	374	393	377	394	378	1.33	4	0	0	0
393	392	375	394	378	395	379	2.43	8	0	2	0
394	393	376	395	379	396	380	2.02	8	0	1	0
395	394	377	396	380	397	381	1.58	6	0	1	0
396	395	378	397	381	398	382	1.74	7	2	0	0
397	396	379	398	382	399	383	1.21	2	0	0	0
398	397	380	399	383	400	384	2.59	9	1	1	0
399	398	381	400	384	401	385	3.23	9	2	0	1
400	399	382	401	385	402	386	1.77	5	1	0	1
401	400	383	402	386	403	387	4.14	12	0	0	0
402	401	384	403	387	404	388	1.51	6	0	0	0
403	402	385	404	388	405	389	1.13	2	0	0	0
404	403	386	405	389	406	390	1	0	0	0	0
405	404	387	406	390	407	391	3.05	8	0	0	0
406	405	388	407	391	408	392	1.23	4	0	0	0
407	406	389	408	392	409	393	1.16	2	0	0	0
408	407	390	409	393	410	394	1.56	5	0	0	0
409	408	391	410	394	411	395	2.36	8	0	0	0
410	409	392	411	395	412	396	1.88	7	2	0	1
411	410	393	412	396	413	397	1.7	6	0	1	0
412	411	394	413	397	414	398	1.39	3	0	2	0

413	412	395	414	398	415	399	1.34	2	2	0	0
414	413	396	415	399	416	400	1	0	1	0	0
415	414	397	416	400	417	401	1.92	8	1	0	0
416	415	398	417	401	418	402	12.97	14	0	0	0
417	416	399	418	402	419	403	1.48	6	0	0	0
418	417	400	419	403	420	404	1.34	2	7	0	0
419	418	401	420	404	421	405	2.11	8	0	0	0
420	419	402	421	405	422	406	1.39	3	4	0	0
421	420	403	422	406	423	407	2.08	9	1	0	0
422	421	404	423	407	424	408	1.46	4	0	0	0
423	422	405	424	408	425	409	2.48	8	0	0	1
424	423	406	425	409	426	410	1.56	4	0	0	0
425	424	407	426	410	427	411	1.11	2	1	0	0
426	425	408	427	411	428	412	2.75	11	0	0	2
427	426	409	428	412	429	413	1.11	2	0	0	0
428	427	410	429	413	430	414	4.1	10	0	0	0
429	428	411	430	414	431	415	1.28	4	1	0	0
430	429	412	431	415	432	416	1	0	0	0	0
431	430	413	432	416	433	417	1.69	5	0	0	0
432	431	414	433	417	434	418	1.51	5	0	0	0
433	432	415	434	418	435	419	3.04	11	0	0	0
434	433	416	435	419	436	420	1.11	2	0	1	0
435	434	417	436	420	437	421	2.73	10	1	0	0
436	435	418	437	421	438	422	1	0	0	0	0
437	436	419	438	422	439	423	1.11	2	0	0	0
438	437	420	439	423	440	424	1	0	0	0	0
439	438	421	440	424	441	425	1.65	3	0	0	0
440	439	422	441	425	442	426	1.67	3	1	0	0
441	440	423	442	426	443	427	1.66	4	0	0	0
442	441	424	443	427	444	428	1.33	3	0	0	0
443	442	425	444	428	445	429	1.68	3	0	0	0
444	443	426	445	429	446	430	1.6	6	0	0	0
445	444	427	446	430	447	431	1.37	5	0	0	0
446	445	428	447	431	448	432	1.06	1	0	0	1
447	446	429	448	432	449	433	1	0	0	0	0
448	447	430	449	433	450	434	1	0	0	0	0
449	448	431	450	434	451	435	1.11	2	0	0	0
450	449	432	451	435	452	436	3.03	10	7	2	1
451	450	433	452	436	453	437	1.36	3	8	0	0
452	451	434	453	437	454	438	1.94	5	0	0	0
453	452	435	454	438	455	439	1.73	4	0	0	0
454	453	436	455	439	456	440	3.26	11	1	0	0
455	454	437	456	440	457	441	1.23	3	0	2	0
456	455	438	457	441	458	442	1.79	6	6	0	0
457	456	439	458	442	459	443	2.33	9	2	0	0
458	457	440	459	443	460	444	1.11	2	0	2	0

459	458	441	460	444	461	445	1.37	4	1	1	0
460	459	442	461	445	462	446	11.07	13	2	3	0
461	460	443	462	446	463	447	3.03	9	2	0	0
462	461	444	463	447	464	448	1	0	0	0	1
463	462	445	464	448	465	449	1.49	5	1	0	0
464	463	446	465	449	466	450	1.54	5	0	0	0
465	464	447	466	450	467	451	5.67	13	1	0	0
466	465	448	467	451	468	452	1.34	6	1	0	0
467	466	449	468	452	469	453	2.75	9	1	1	0
468	467	450	469	453	470	454	5.26	10	2	0	0
469	468	451	470	454	471	455	1.47	5	0	0	0
470	469	452	471	455	472	456	1	0	0	0	0
471	470	453	472	456	473	457	6.05	11	2	0	0
472	471	454	473	457	474	458	1.52	6	1	0	0
473	472	455	474	458	475	459	1	0	0	0	0
474	473	456	475	459	476	460	1	0	0	0	0
475	474	457	476	460	477	461	1.9	6	0	0	0
476	475	458	477	461	478	462	2.88	7	0	0	0
477	476	459	478	462	479	463	10.46	11	0	0	0
478	477	460	479	463	480	464	1.05	1	1	0	0
479	478	461	480	464	481	465	2.59	6	1	0	0
480	479	462	481	465	482	466	1.39	2	2	0	0
481	480	463	482	466	483	467	1	0	0	0	0
482	481	464	483	467	484	468	1	0	0	0	0
483	482	465	484	468	485	469	2.16	8	0	0	0
484	483	466	485	469	486	470	1.11	2	0	0	0
485	484	467	486	470	487	471	1.73	8	0	0	0
486	485	468	487	471	488	472	1	0	0	0	0
487	486	469	488	472	489	473	2.2	7	0	0	0
488	487	470	489	473	490	474	1	0	0	0	0
489	488	471	490	474	491	475	2.44	8	1	0	0
490	489	472	491	475	492	476	2.39	10	0	0	0
491	490	473	492	476	493	477	9.71	14	0	0	0
492	491	474	493	477	494	478	1	0	0	0	0
493	492	475	494	478	495	479	2.21	8	1	0	0
494	493	476	495	479	496	480	4.72	10	0	0	0
495	494	477	496	480	497	481	1.55	6	0	0	0
496	495	478	497	481	498	482	3.51	13	0	0	0
497	496	479	498	482	499	483	1.48	6	1	0	0
498	497	480	499	483	500	484	1.16	2	0	0	0
499	498	481	500	484	501	485	2.34	9	2	1	1
500	499	482	501	485	502	486	1.48	4	0	0	0
501	500	483	502	486	503	487	1	0	0	0	0
502	501	484	503	487	504	488	3.09	12	1	0	0
503	502	485	504	488	505	489	1.12	2	0	0	0
504	503	486	505	489	506	490	2.94	8	0	0	2

505	504	487	506	490	507	491	4.9	10	0	0	0
506	505	488	507	491	508	492	1	0	0	0	0
507	506	489	508	492	509	493	1.78	5	0	0	0
508	507	490	509	493	510	494	2.35	11	2	0	1
509	508	491	510	494	511	495	1.48	4	0	0	0
510	509	492	511	495	512	496	2.2	9	0	0	0
511	510	493	512	496	513	497	2.54	10	0	0	0
512	511	494	513	497	514	498	1.69	6	0	1	0
513	512	495	514	498	515	499	4.29	13	0	0	0
514	513	496	515	499	516	500	1.61	6	0	0	0
515	514	497	516	500	517	501	2.16	10	0	0	0
516	515	498	517	501	518	502	3.49	12	0	0	0
517	516	499	518	502	519	503	1.44	4	0	0	0
518	517	500	519	503	520	504	2.9	12	2	0	1
519	518	501	520	504	521	505	1.71	3	1	0	2
520	519	502	521	505	522	506	1.36	4	0	0	0
521	520	503	522	506	523	507	2.3	9	0	0	0
522	521	504	523	507	524	508	1	0	0	0	0
523	522	505	524	508	525	509	2.2	9	0	0	0
524	523	506	525	509	526	510	1.56	6	0	1	0
525	524	507	526	510	527	511	4.11	13	0	0	0
526	525	508	527	511	528	512	2.87	12	0	0	0
527	526	509	528	512	529	513	4.81	14	0	0	0
528	527	510	529	513	530	514	2.34	8	0	0	1
529	528	511	530	514	531	515	1.82	6	0	0	0
530	529	512	531	515	532	516	2.03	5	0	0	0
531	530	513	532	516	533	517	1.36	3	0	0	0
532	531	514	533	517	534	518	1.38	4	0	0	1
533	532	515	534	518	535	519	1.06	1	0	0	0
534	533	516	535	519	536	520	1.11	2	0	0	0
535	534	517	536	520	537	521	1.49	4	0	0	0
536	535	518	537	521	538	522	1.89	6	0	0	0
537	536	519	538	522	539	523	1.38	6	0	0	0
538	537	520	539	523	540	524	1.11	2	0	0	0
539	538	521	540	524	541	525	1.67	5	0	0	0
540	539	522	541	525	542	526	1.49	5	0	0	1
541	540	523	542	526	543	527	1.27	2	0	0	0
542	541	524	543	527	544	528	2.33	7	0	0	1
543	542	525	544	528	545	529	1.68	5	0	0	0
544	543	526	545	529	546	530	3.4	11	1	0	0
545	544	527	546	530	547	531	1.57	6	0	0	0
546	545	528	547	531	548	532	3.23	10	0	0	0
547	546	529	548	532	549	533	2.77	9	0	0	0
548	547	530	549	533	550	534	1	0	0	0	0
549	548	531	550	534	551	535	1.85	6	0	0	0
550	549	532	551	535	552	536	3.54	9	0	0	0

551	550	533	gap	gap	553	537	2.61	10	0	0	0
552	551	534	552	536	554	538	2.94	11	0	0	0
553	552	535	553	537	555	539	1	0	0	0	0
554	553	536	554	538	556	540	2.06	6	0	0	0
555	554	537	555	539	557	541	1	0	0	0	0
556	555	538	556	540	558	542	1.39	5	0	0	0
557	556	539	557	541	559	543	3.62	6	0	0	0
558	557	540	558	542	560	544	1.34	2	0	0	0
559	558	541	559	543	561	545	2.78	7	0	0	0
560	559	542	560	544	562	546	1.5	4	0	0	0
561	560	543	561	545	563	547	1.72	6	0	0	0
562	561	544	562	546	564	548	1	0	0	1	0
563	562	545	563	547	565	549	1.5	5	0	1	0
564	563	546	564	548	566	550	2.72	10	1	0	0
565	564	547	565	549	567	551	1	0	0	0	0
566	565	548	566	550	568	552	1.34	2	0	0	0

Suppl. Table 2. HA numbering conversion table for influenza subtypes H1, H3 and H5. The eighth column is the geometric mean of Odds Ratios for all the influenza A subtypes and passage categories combinations examined. The last 3 columns (#ssdp H1p, #ssdp H1s, #ssdp H3) indicate the number of SSDP strains where mutations were found at the corresponding sites. Passage bias sites were identified using (i) same strain different passage (SSDP) analysis, (ii) large-scale analysis of sequence and passage data until May 2013, and (iii) large-scale analysis for sequence and passage data from June 2013 to May 2019. The 19 passage bias sites that are found in all 3 analyses are highlighted in pink. The remaining 35 passage bias sites found in at least 2 out of 3 of the above mentioned analyses are highlighted in green.

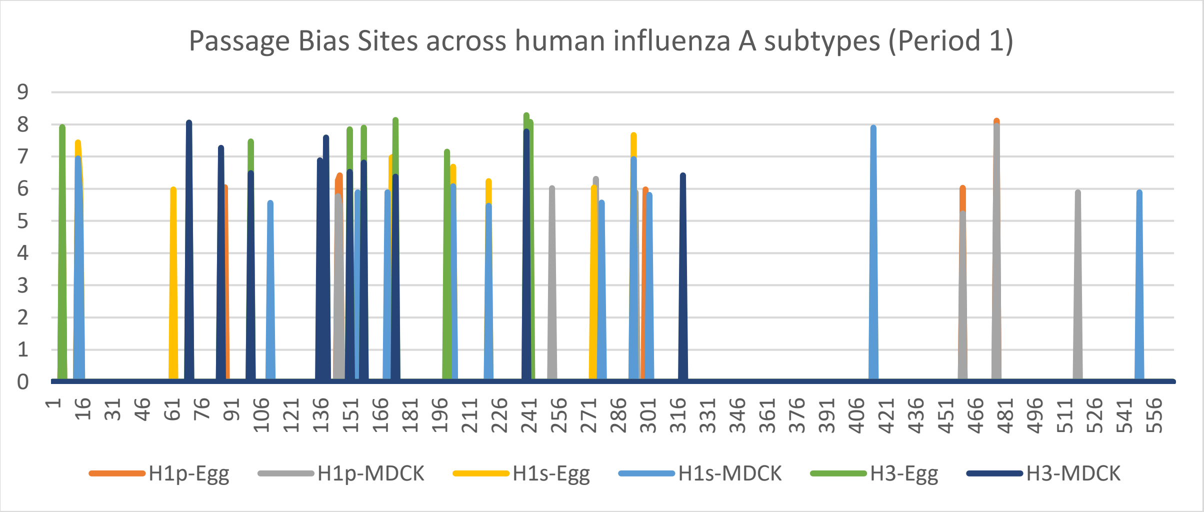

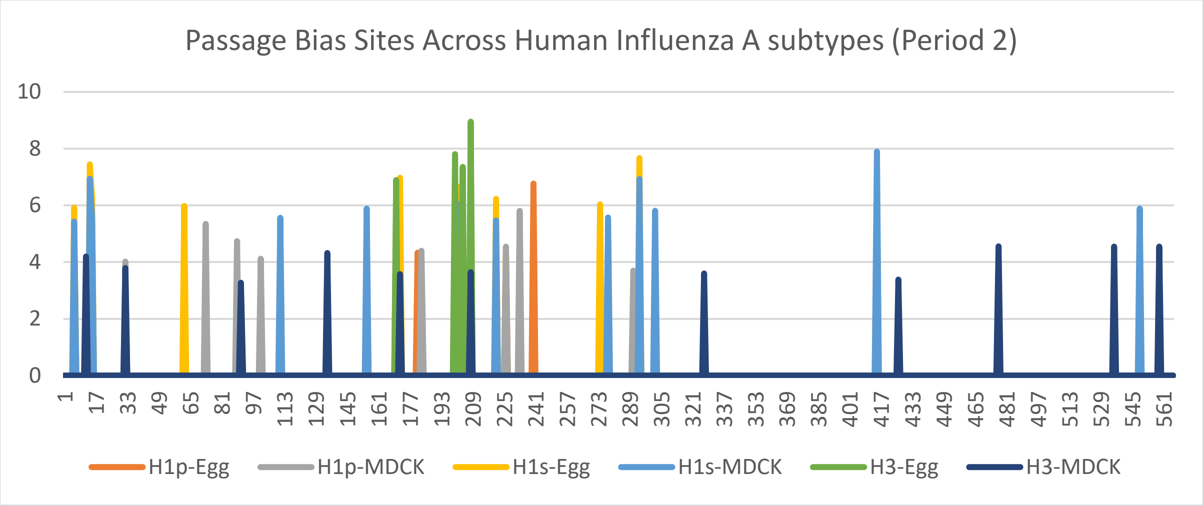

Suppl. Fig. 2. Distribution of passage bias sites across the HA sequence comparing data from two time periods. The graphs show the top 5 percent log odds ratios of passage bias sites from MDCK cells and Egg cells for the human seasonal influenza viruses. For the 63 passage bias sites found in both time periods, 54 of them have geometric mean values that are higher than 3.6 (last column in Suppl. Table 2)

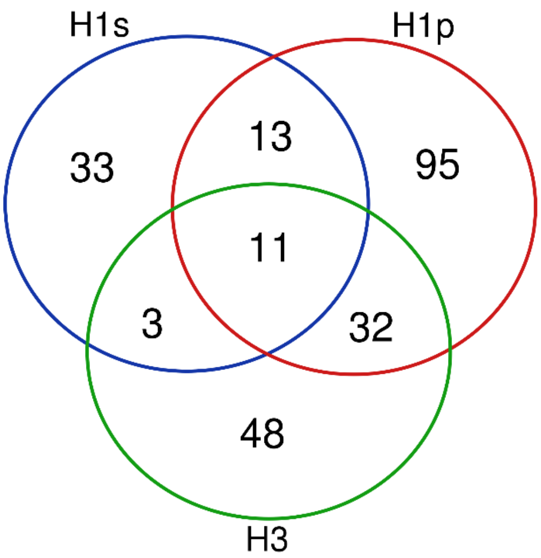

Suppl. Fig. 3. Overlap of SSDP (same strain different passage) sites between the 3 seasonal subtypes.

SetA	SetB	overlap-pos (H1pdm absolute numbering)	#overlap-pos	#SetA-pos	#SetB-pos	OddR
SSDP	H5inv	151|173|200|204|207|210|236|240|241|269	10	59	21	9.202226

<table>
<tr><td></td><td></td><td></td><td></td><td></td><td></td><td></td></tr>
<tr><td>ODDS-T2</td><td>H5inv</td><td>111|173|200|201|207|210|240</td><td>7</td><td>52</td><td>21</td><td>5.555556</td></tr>
<tr><td>ODDS-T1</td><td>H5inv</td><td>111|151|157|173|200|201|206|207|210|236|240|241|253</td><td>13</td><td>82</td><td>21</td><td>11.21014</td></tr>
<tr><td>overlap>=2sets</td><td>H5inv</td><td>111|151|173|200|201|207|210|236|240|241</td><td>10</td><td>54</td><td>21</td><td>10.35124</td></tr>
<tr><td>overlap>=3sets</td><td>H5inv</td><td>173|200|207|210|240</td><td>5</td><td>19</td><td>21</td><td>11.85268</td></tr>
</table>

Suppl. Table 3. Overlap of selected sets with WHO H5 genetic change inventory

	H1p	H1s	H3	H5 (human)	H5 (avian)
H120Y	Insufficient data	No data for residue pair	Insufficient data	insufficient data	Insufficient data
N172D	No data for residue pair	D preferred in Egg. Wrong Prediction. Odds Ratio 18.0	D preferred in MDCK. Correctly Predicted. Odds Ratio 8.21	Weak Odds Ratio close to 1.	N preferred in Egg. Correctly Predicted. Odds Ratio 2.54
T174A	A preferred in MDCK. Correctly Predicted. Odds Ratio 2.13.	No data for residue pair	A preferred in Egg. Wrong Prediction. Odds Ratio 3129	A preferred in ORI. Correctly Predicted. Odds Ratio 4.	T preferred in Egg. Correctly Predicted. Odds Ratio 4.31
N238K	No data for residue pair	Insufficient data.	Insufficient data	Insufficient data	Insufficient data
Q240L	No data for residue pair	No data for residue pair	Q preferred in Egg. Correctly Predicted. Odds Ratio 514.	Insufficient data	Insufficient data
G242S	No data for residue pair	Insufficient data	G preferred in Egg. Correctly Predicted. Odds Ratio 11069.	Insufficient data	Insufficient data
T333I	Insufficient data	Insufficient data	I preferred in Egg. Wrong Prediction. Odds Ratio 26.8.	Insufficient data	Insufficient data

Suppl. Table 4. Prediction of avian to mammalian adaptation mutations for the 7 mutations identified in the gain-of-ferret transmissibility studies using large scale sequence analysis of passage bias. To see if these 7 mutations could be correctly predicted using viral sequence and passage data from H5 (avian) viruses, we first look at the Odds Ratio for the amino acid preference in Egg versus mammalian cells (MDCK or ORI). For 5 out of the 7 mutations, there were insufficient data for H5

(avian) viruses because most of these viruses are grown in Egg cells (Table 1). For N172D and T174A, the H5 (avian) sequence and passage data correctly predicted the amino acid preference. Next, we used passage bias data from human viruses to see if we can predict the rest of the mutations correctly. Passage bias data derived from H3 viruses correctly predicted the amino acid preference for Q240L and G242S but wrongly predicted the direction of the T333I mutation. Amino acid preference data is insufficient for the remaining 2 mutations (H120Y and N238K).
